# Artificial Intelligence in Rheumatology: Quo Vadis?

**DOI:** 10.31138/mjr.160825.erh

**Published:** 2025-09-30

**Authors:** Alexandra Ainatzoglou

**Affiliations:** Fourth Department of Internal Medicine, Hippokration Hospital, Thessaloniki, Greece

**Keywords:** artificial intelligence, rheumatology, machine learning, AI, RMD, KAP

Over the past few years, medical applications of artificial intelligence (AI) have seen exponential growth, introducing innovative tools that revolutionised formerly established practices. The advent of AI has been disseminated in the field of rheumatic and musculoskeletal diseases (RMDs), marking a new era in the diagnosis and management of rheumatic patients. This research topic for the Mediterranean Journal of Rheumatology touches upon this ongoing evolution, comprising two articles by distinguished contributors; a viewpoint authored by Pelechas et al.^1^ delineating the challenges and limitations involved in the integration of AI in rheumatologic settings and the original study of Mahadevan et al.^2^ palpating the current knowledge, attitude, and practices (KAP) of rheumatologic professionals.

The core capacity of AI to emulate human cognition and reasoning allows it to augment human intelligence in health-related problem-solving and guide informed decision-making.^3^ Machine learning (ML) is an AI subset designed to generate predictive models after receiving training on input data. Opposing to conventional statistics, ML facilitates pattern recognition and uncovers novel correlations, promoting hypothesis generation, while enabling big data processing. This capacity is required in genomic and transcriptomic analyses^4^ necessary for personalised medicine, where AI methods can be leveraged to optimise diagnostic and therapeutic practices.

Inter alia, AI promises to enhance medical training, facilitate the digitalisation of health records,^5^ automate medical imaging analysis, promote early diagnosis through biomarker discovery, and guide tailored drug development.^6^ Examples include the use of deep learning to structure ultrasound-derived images for sonographer education^7^ and the assessment of osteophytes using AI models trained on ultrasound imaging.^8^ Telemedicine has also been accelerated through convolutional neural networks, inferring joint inflammation in RA through smartphone images.^9^ Such digital biomarkers predicting flare risk through wearable data analysis^15^ enable constant patient monitoring and orient treatment decisions.^33^

Although most of these pledges are already underway, the speedy adoption of ground-breaking technologies in any healthcare ecosystem does not come unhindered.^10^ In this issue’s viewpoint, Pelechas et al.^1^ critically revisit the pitfalls preventing AI from substituting rheumatological expertise. Firstly, most RMDs present with a wide range of symptoms, often non-scalable, impeding the application of AI methods eligible for homogenous data.^11^ In contrast with radiology and pathophysiology that highly encourage AI-assisted image mapping,^12,13^ rheumatology necessitates clinician judgment in all diagnostic and therapeutic processes. Authors further argue that shared decision-making is crucial in rheumatic patient care, requiring soft skills such as combinative reasoning that AI cannot replicate, solely complement.^14^

Concerns still remain on privacy issues, model interpretability, regulatory frame^15^ and technical barriers on computational power.^16^ Notably, most RMDs lack a definitive diagnostic test, while tests commonly used in rheumatic patients are highly susceptible to false positivity or negativity, limiting their predictive value. Adding to that, established rheumatologic criteria mostly assist in disease classification rather than diagnosis, while numerous patients present with atypical disease phenotypes. Authors underline that RMDs with manifestations of varying severity and conflicting test results pose diagnostic challenges calling for interpretation of contextual factors and subjective physician judgment, perplexing diagnostic automatisation.^17^

Along the same lines, the therapeutic responses of rheumatic patients remain largely heterogenous, rendering treatment algorithms particularly intriguing. ML models require clean data as input and yield standardised approaches, hard to apply in non- “one-size-fits-all” RMDs. Pelechas et al.^1^ further denote that training AI models on research findings is prone to fallacy, as study outcomes are subject to bias and methodological errors, often deriving from restricted samples.^18^ Such models can only be as reliable as the data they get trained on, rendering inherent bias inevitable despite system training advancements.^19^ Given their lack of transparency, authors reckon that correcting flawed models can be hard.^20^ To prevent errors that could erode clinician trust, authors suggest rigorous algorithm validation on real-world data and constant monitoring.

Nonetheless, rapidly evolving AI modalities have already addressed the aforementioned challenges, substantially assisting rheumatic care. Regarding intricate diagnostics, a physician-friendly ML-based algorithm was developed to facilitate early SLE diagnosis,^21^ displaying excellent efficacy^20,21^ and comparable accuracy with established classification criteria.^24^ Moreover, AI has guided several attempts to decipher the molecular landscape of rheumatic patients. Transcriptomic profiling and ML have been deployed to pinpoint patient endotypes with unique gene expression profiles reflecting varying clinical severity,^25–27^ while a signature predicting disease activity based on AI-mediated transcriptomics paved the way for liquid biopsy.^28^ Passing to therapeutics, endotypes were further associated with treatment response to Belimumab^29^ and investigational compounds,^25,30,31^ while predictive models decoded RA patient response to methotrexate and biologics.^32^

These AI-powered leaps in understanding the molecular taxonomy underlying symptom variability and treatment response heterogeneity of rheumatic patients were once unimaginable. To grasp the sentiment of rheumatologists towards AI, Mahadevan et al.^2^ assessed their KAP cross-sectionally through questionnaire completion. Physician awareness on AI technology was evaluated at satisfactory levels, yet their actual knowledge was limited, with a difficult learning curve owing to training deficits. Suboptimal clinical implementation of AI tools was principally attributed to privacy and ethical concerns. Despite fear of expertise loss, the overall sentiment remained positive, as most participants accredited the potential of AI to enhance health outcomes of RMDs. These findings align with reports documenting that 73% of rheumatologists never utilised AI in clinical practice and with salutary health outcomes registered in AI-mediated approaches.^34^

Taken together, this issue underscores the overall stance of rheumatology professionals on the dissemination of AI in their field of expertise and their belief in its transformative impact on various aspects of patient care. Further, it provides insight into the pitfalls inherent in the adoption of this novel technology. To address these caveats, a multidisciplinary approach involving rheumatologists, AI specialists, industry stakeholders and regulatory bodies is warranted to safeguard the equitable integration of AI methods in rheumatology,^34^ while endeavours of clinician education should be prioritised, promoting the widespread yet ethical use of this advent.^35^

## CONFLICT OF INTEREST

The authors declare no conflict of interest.
